# Distal triceps tendon repair using Krakow whipstitches, K wires, tension band and double drilling technique: a case report

**DOI:** 10.1186/s13256-014-0504-5

**Published:** 2015-02-19

**Authors:** Luigi Tarallo, Francesco Zambianchi, Raffaele Mugnai, Carlo Alberto Costanzini, Fabio Catani

**Affiliations:** Department of Orthopaedics and Traumatology, University Hospital Policlinico di Modena, Via del Pozzo 71, 41124 Modena, Italy

**Keywords:** Double drilling, K wires, Olecranon, Suture, Triceps tendon

## Abstract

**Introduction:**

The management of distal triceps tears must address each patient’s medical and functional status: in general, the literature has described satisfactory nonsurgical treatment in tears less than 50%. Tears greater than 50% are treated nonsurgically in a sedentary person and surgically in active patients. Complete tears are generally managed surgically: most reported repair techniques describe the use of Bunnell or Krakow whipstitch techniques, passing the sutures through transosseous drill holes in the ulna. Other described techniques include the use of suture anchors and direct tendon repair to a periosteal flap raised from the olecranon.

**Case presentation:**

In the presented report we describe the surgical technique used to treat a complete traumatic distal triceps tendon rupture associated with olecranon fracture in a 40-year-old Caucasian man with underlying poor tendon quality and postoperative assessment. To the best of our knowledge no studies describing the performed surgical technique, utilizing Krakow whipstitches, olecranon fixation with K wires and Zuggurtung tension band through transosseous drill holes have been previously described in the literature.

At 30 days postoperatively the patient had regained full elbow flexion/extension and pronation/supination.

**Conclusions:**

The described methodology, using a double ulnar tunnel to obtain fixation of the fragment, associated with a whipstitch locking-type suture for the triceps tendon, allowed proper fixation of the fracture and optimal reinsertion of the detached tendon on its footprint with sufficient strength.

## Introduction

Distal rupture of the triceps tendon represents a rare injury and is described as the least common of reported tendon ruptures [1]. Most reports regarding distal triceps tendon rupture have associated the injury with anabolic steroid use, weight lifting and violent sport practice. Other factors that may play a role in distal triceps rupture include local steroid injection, metabolic bone diseases (i.e. hyperparathyroidism), renal osteodystrophy, olecranon bursitis hypocalcemic tetany, Marfan syndrome, osteogenesis imperfecta, rheumatoid arthritis and type I diabetes [2-5].

The most commonly described injury mechanism is a sudden eccentric load applied to a contracting triceps muscle, such as a fall onto an outstretched hand or during weight lifting. Lacerations and open injuries can also cause triceps tendon rupture: a direct blow may result in such a lesion, even though this presentation is less common. High-energy mechanisms, such as vehicle accidents, can also be responsible for ulnar nerve impairment and compartment syndrome [5].

The diagnosis of distal triceps rupture is generally guided by clinical examination and radiologic imaging. A modified Thompson squeeze test, similar in execution to that performed for Achilles tendon, has been reported as a potential clinical diagnostic tool. Patients with incomplete triceps rupture will be able to extend their elbow against gravity after triceps muscle squeezing by the examiner. Patients with complete disruption of their triceps proper and lateral expansion will not be able to extend their triceps against gravity. A lateral radiograph may show the presence of flecks of avulsed osseous material from the olecranon, which is almost pathognomonic of a triceps tendon rupture. A computed tomography scan can be helpful in excluding associated bony injuries. Magnetic resonance imaging (MRI) and ultrasounds are useful in cases in which it is hard to define whether the lesion is complete or partial.

Management of distal triceps tears must address each patient’s medical and functional status: in general, the literature has described satisfactory nonsurgical treatment in tears less than 50% [6]. Tears greater than 50% are treated nonsurgically in a sedentary person and surgically in active patients [2]. Complete tears are generally managed surgically: most reported repair techniques describe the use of Bunnell or Krakow whipstitch techniques, passing the sutures through transosseous drill holes in the ulna, as reported by van Riet *et al*. [7], resulting in 21% rate of rerupture of the tendon. Other described techniques include the use of suture anchors and direct tendon repair to a periosteal flap raised from the olecranon [5]. Usual postoperative care includes immobilization in 30° to 45° of flexion for two weeks. One month postoperatively, active range of motion (ROM) is initiated and efforts are focused on full elbow motion regain. Weight lifting is discouraged for at least four to six months postoperatively. Results following primary repair of acute triceps injuries are very good. Most published reports do not provide quantitative or subjective outcome data based on the Disabilities of the Arm, Shoulder and Hand measure or Mayo Elbow Performance score Most studies offer a retrospective review of cases performed and state that the patients demonstrate good ROM and strength with return to previously performed levels of function.

In the presented report we describe the surgical technique used to treat a complete traumatic distal triceps tendon rupture associated with olecranon fracture in a 40-year-old man with underlying poor tendon quality and postoperative assessment. To the best of our knowledge, no reports describing the performed surgical technique, utilizing Krakow whipstitches, olecranon fixation with K wires and Zuggurtung tension band through transosseous drill holes have been previously described in the literature.

## Case presentation

**Figure 1:**
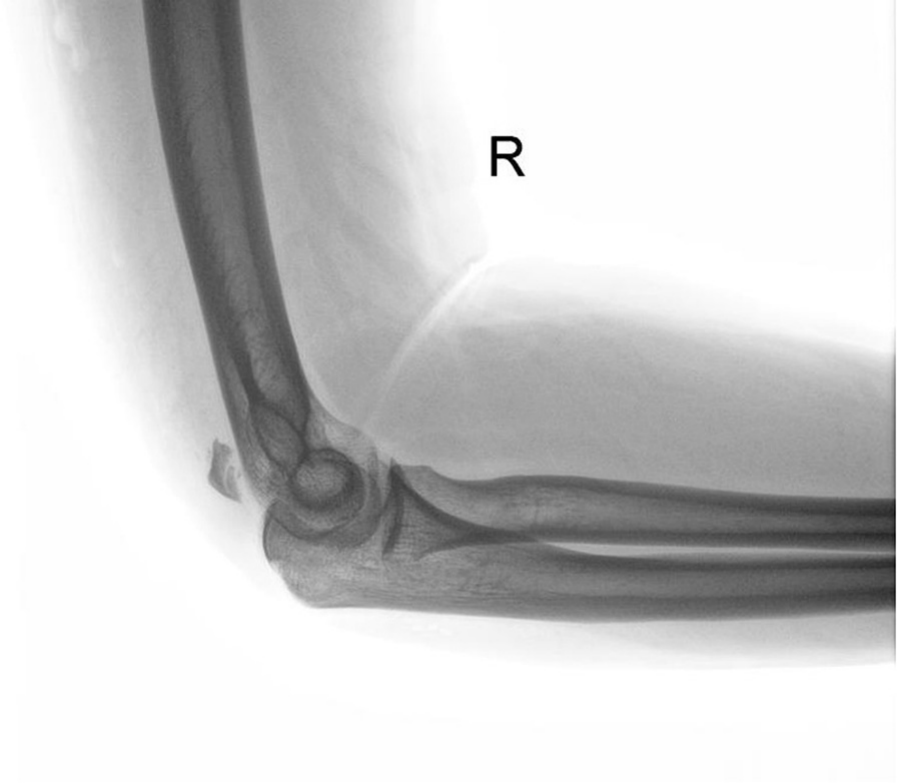
**Preoperative radiograph showing proximal olecranon avulsion.**

A 40-year-old Caucasian man presented at our emergency department after a high-energy car accident, complaining of pain to his right elbow and on the medial side of his right hand. A clinical examination revealed tenderness to palpation and swelling. He was unable to actively reach full extension of his forearm. Radiographic examination revealed complete avulsion of the proximal aspect of his olecranon (Figure 1) and displaced fracture of the base of his fifth metacarpal bone. Ultrasonography examination was performed, which showed complete triceps tendon avulsion from the dislocated olecranon fragment. He underwent surgery for triceps tendon relocation, open reduction and fixation of the olecranon avulsion and fixation of his fifth metacarpal bone two days after the injury.

**Figure 2:**
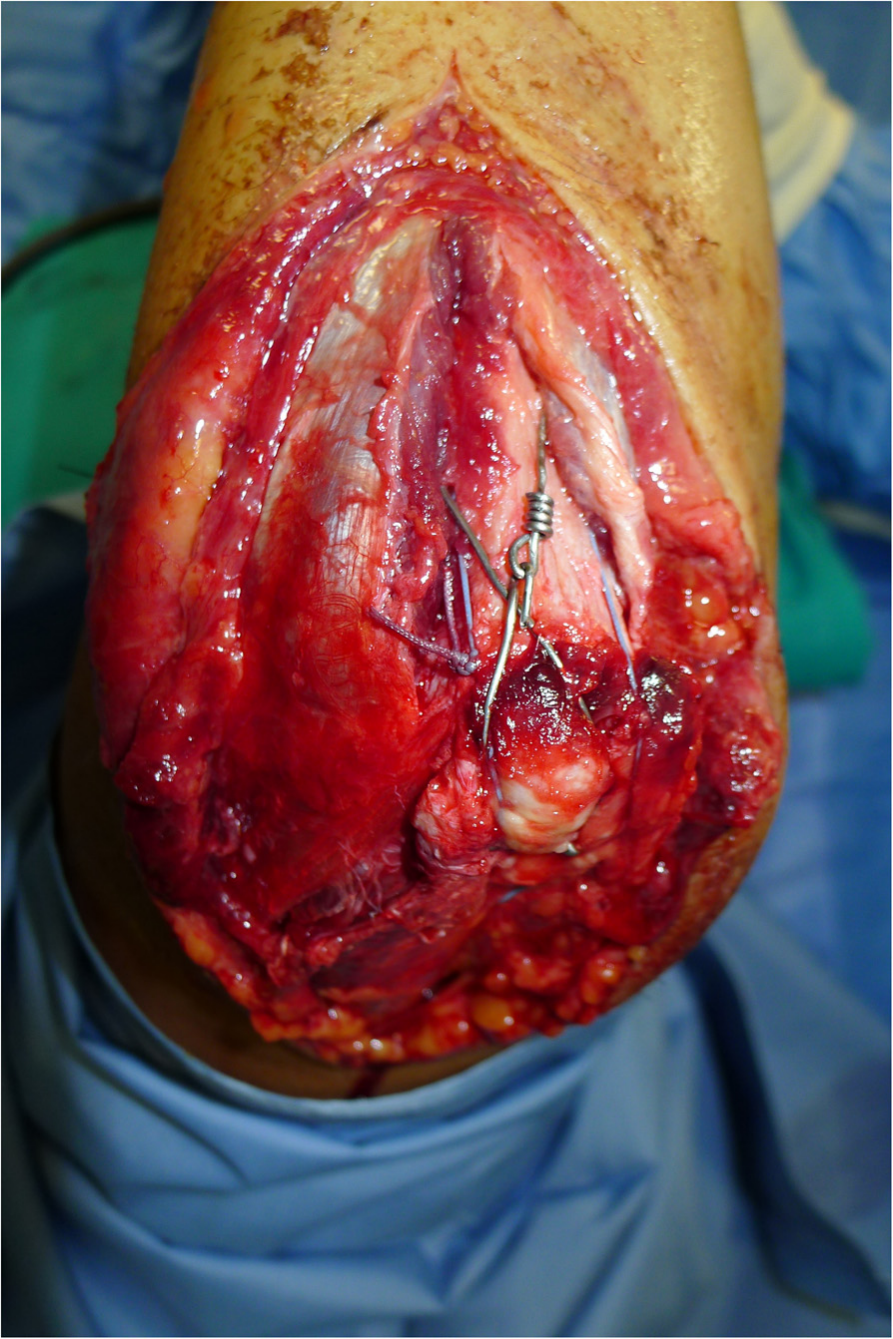
**Intraoperative fixation of the olecranon fragment with K wires tension band and triceps tendon reinsertion with Krakow sutures.**

**Figure 3:**
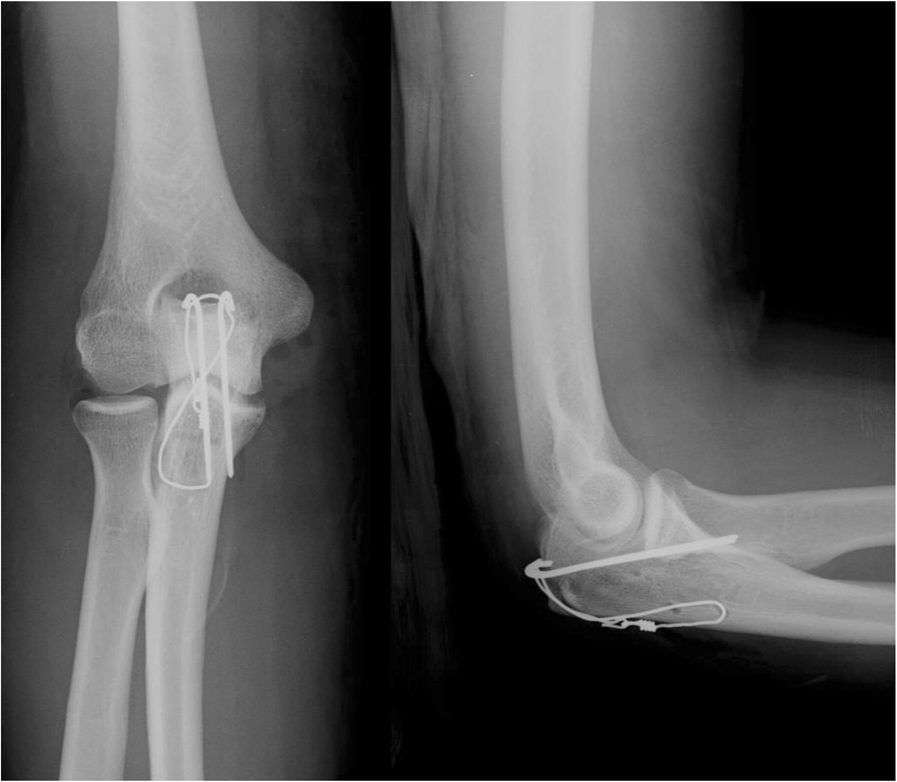
**Postoperative radiographs showing the double drilling in the proximal part of the ulna.** Olecranon avulsion fixed with a Zuggurtung tension band technique.

He lay in a supine position with his right arm in a flexed position. His upper extremity was draped free so that his elbow could be manipulated comfortably. A posterior surgical approach, which consisted of a Z incision over his elbow, was used so that wound healing was not hindered by postoperative ROM, as the tip of the olecranon becomes more prominent during elbow flexion. The exposure was made distal enough to visualize the entire insertion of the tendon over the ulnar surface. His ulnar nerve was identified and tied. The olecranon bony insertion site was excoriated to remove interposed soft tissues. An intraoperative examination revealed that his olecranon was displaced and divided into two fragments. A locking-type whipstitch according to Krakow technique was placed on the tendon with nonabsorbable sutures and four throws were placed on each side. Using fluoroscopy, the displaced olecranon fracture was reduced and fixated with two 1.6 K wires. The sutures were then passed through a transosseous drill hole 2cm distal to the tip of the fixated olecranon fragment (to avoid joint penetration) and subsequently tied over a bone bridge. His arm was held in approximately 35° to 40° of flexion for optimal tensioning and the sutures were tied. Another transosseous drill hole was performed in an oblique way 3cm distal to the first drill hole and using Zuggurtung tension bands, a metal wire was passed under the previously placed K wires on the olecranon. Tension was held on the sutures to reduce the triceps tendon to its desired position on the footprint (Figure 2 and Figure 3).

Postoperative treatment did not include splinting; rather, a sling was used for his comfort. He was allowed to discontinue use of the sling when he felt comfortable. Active motion exercises, including pronation and supination, were begun immediately. He was left free to hang the arm against gravity as long as tolerated. No weight bearing was permitted for the first four weeks postoperatively. Strengthening was begun four weeks postoperatively. At 30 days postoperatively he had regained full elbow flexion/extension and pronation/supination.

## Discussion

Rupture of the triceps tendon is the least common of all tendon injuries [1], occurring after active muscle contraction in extension with a forced passive flexion. Factors such as the use of anabolic steroids and local injections of corticosteroids can increase risk of rupture following minor trauma [2-4]. High-energy traumas applied to the olecranon process can also determine bony avulsion of the tip of the ulnar process, resulting in distal triceps tendon detachment [5]. Clinical assessment is fundamental to diagnose the rupture. Imaging (plain radiographs, ultrasounds and MRI) is important in the diagnosis and preoperative assessment to detect the partial forms avoiding the change into chronic forms. Treatment depends on the extent of tendon rupture, preoperative level of activity performed by the patient and his or her age. Surgical repair remains controversial in partial tendon ruptures and often good results are obtained with conservative treatment: surgical repair has been the mainstay of treatment for most acute tendon ruptures in active patients [8]. Several surgical techniques have been proposed for triceps tendon reinsertion, such as the use of anchors, tendon grafts flaps and locking sutures through the tendon edges passed through three drill holes in the olecranon. To the best of our knowledge, the reported surgical technique has not been described in the literature for avulsion of the proximal tip of the olecranon associated with triceps tendon rupture: the use of a double ulnar tunnel to obtain fixation of the olecranon fragment with Zuggurtung tension bands with K wires, associated with a whipstitch locking-type suture for the triceps tendon was demonstrated to be an effective technique to obtain proper fixation of the fracture and optimal reinsertion of the detached tendon on its footprint. The clinical result was satisfactory with full elbow ROM recovery at 30 days postoperatively.

## Conclusions

The authors recommend surgical tendon repair in young and active patients: the described surgical technique represents a possibility when avulsion of the tip of the olecranon is present.

## Consent

Written informed consent was obtained from the patient for publication of this case report and accompanying images. A copy of the written consent is available for review by the Editor-in-Chief of this journal.

## Abbreviations

MRI: Magnetic resonance imaging
ROM: Range of motion
